# Influence of blood flow restriction cuff width on acute metabolic, perceptual, and landing responses to low-load resistance exercise in trained long jump athletes

**DOI:** 10.3389/fphys.2026.1912080

**Published:** 2026-09-11

**Authors:** Chen Wang, Huizhen Yang

**Affiliations:** School of Education, Beijing Sport University, Beijing, China

**Keywords:** arterial occlusion pressure, blood flow restriction, blood lactate, cuff width, rating of perceived exertion

## Abstract

**Background:**

Blood flow restriction (BFR) is used with low-load resistance exercise, but acute responses may depend on cuff width when pressure is prescribed relative to arterial occlusion pressure (AOP).

**Aim:**

To compare acute metabolic, perceptual, and landing-related responses among No-BFR, 6-cm BFR, and 12-cm BFR conditions in trained male long-jump athletes.

**Methods:**

Twenty-two athletes completed the three conditions in a randomized crossover design. Participants performed Smith-machine half squats at 30% of one-repetition maximum using four sets of 30, 15, 15, and 15 repetitions with 30-s inter-set rest. Cuffs were maintained at 40% of cuff-specific AOP. Blood lactate was measured before, immediately after, and 5 min after exercise. Rating of perceived exertion (RPE) and numerical pain rating scale (NPRS) scores were recorded after each set. Drop-jump (DJ) height, loading rate, and center-of-pressure (COP) sample entropy were measured before and after exercise.

**Results:**

BFR produced higher blood lactate, RPE, and NPRS than No-BFR, particularly during later assessments. Significant condition-by-time/set interactions were observed for blood lactate (F(2.24,47.12)=14.101, p<0.001, ηp²=0.402), NPRS (F(4.34,91.04)=4.839, p=0.001, ηp²=0.180), DJ height (F(1.76,37.04)=20.163, p<0.001, ηp²=0.490), loading rate (F(1.87,39.31)=3.850, p=0.032, ηp²=0.155), and COP sample entropy (F(1.51,31.65)=7.521, p=0.004, ηp²=0.264); the RPE interaction was not significant (p = 0.094). Following BFR, DJ height decreased, whereas loading rate and COP sample entropy increased. The 12-cm condition generally produced the largest responses, although direct cuff-width differences were not uniform.

**Conclusions:**

Cuff width influenced acute metabolic, perceptual, and post-exercise landing responses during low-load BFR exercise. These acute findings do not establish superior long-term training adaptations.

## 1 Introduction

Blood flow restriction (BFR) exercise is attractive in sport because low-load resistance exercise combined with BFR can support strength and muscle-mass adaptations (Wortman et al., 2020). This low-load approach may be useful for trained athletes who need to manage joint stress, tissue irritation, residual fatigue, or congested training schedules. Although high-load resistance training remains the foundation of athletic strength development, there are periods when adding more heavy loading may be poorly timed, such as rehabilitation, deloading, return-to-training, or sessions scheduled close to sprinting, jumping, or technical work. In these situations, low-load exercise combined with BFR may provide a useful alternative by increasing local physiological stress at substantially lower external loads (Ohta et al., 2003; Lixandrão et al., 2018). More recent athlete-focused syntheses similarly frame BFR as a useful strategy when training stress must be balanced against performance and recovery demands (Wortman et al., 2021; Li et al., 2024).

The rationale for BFR is that cuff pressure may change the exercising muscle environment, rather than simply making a light exercise feel harder. BFR is generally expected to restrict venous return and partially limit arterial inflow, thereby promoting local hypoxia and metabolite accumulation during low-load contractions (Suga et al., 2009). Metabolites such as lactate, hydrogen ions, and inorganic phosphate may contribute to fatigue development as the exercise bout progresses (Pearson and Hussain, 2015; Nalbandian and Takeda, 2016). BFR-specific reviews and acute studies further describe altered metabolic and fatigue-related responses during low-load resistance exercise (Pope et al., 2013; Wernbom and Aagaard, 2020; Hill et al., 2022). However, these mechanisms should be interpreted as a theoretical framework rather than as processes directly measured in every acute BFR study. Accordingly, blood lactate, rating of perceived exertion (RPE), and numerical pain rating scale (NPRS) scores were included to characterize the acute metabolic and perceptual responses to the exercise protocol. This low-load rationale is also supported by work describing BFR-related metabolic and neuromuscular fatigue responses during resistance exercise (Pope et al., 2013; Wernbom and Aagaard, 2020; Hill et al., 2022).

BFR prescription is not determined by exercise load alone. Applied pressure, cuff width, cuff material, limb circumference, cuff placement, and the mode of pressure maintenance all influence the actual stimulus experienced by the participant. For this reason, contemporary methodological recommendations emphasize individualized pressure prescription based on arterial occlusion pressure (AOP), rather than relying only on a fixed absolute pressure (Patterson et al., 2019). AOP-based prescription is especially important because wider cuffs generally require lower absolute pressures to restrict arterial flow, whereas narrower cuffs require higher pressures to reach a comparable occlusion endpoint (Loenneke et al., 2012; Jessee et al., 2016). However, using relative AOP does not necessarily mean that all cuff widths produce identical physiological or perceptual responses. Recent methodological analyses further caution that relative pressure, cuff size, body position, and practical pressure-estimation methods can materially affect BFR standardization (Clarkson et al., 2020; Aniceto and da Silva, 2022; de Queiros et al., 2024).

Cuff width is therefore not a minor equipment detail. It may shape the spatial distribution of pressure, the degree of tissue compression, the relation between external cuff pressure and intramuscular pressure, and the balance between vascular restriction and perceived discomfort. Hemodynamic studies have shown that cuffs differing in width or design can produce distinct blood-flow responses, even when the pressure is expressed relative to AOP (Mouser et al., 2017; Mouser et al., 2018). Training studies have also suggested that cuff width can influence adaptation or tolerance depending on how pressure is prescribed (Laurentino et al., 2016). This makes cuff width an applied prescription variable with potential consequences for acute metabolic, hemodynamic, and perceptual responses.

The perceptual dimension is particularly important in athletes because a training method that increases physiological stimulus but produces excessive discomfort may reduce compliance, impair technical execution, or interfere with subsequent high-quality work. BFR exercise is accompanied by discomfort that may be influenced by applied pressure, cuff width, cuff material, and exercise design (Soligon et al., 2018; Spitz et al., 2020; Spitz et al., 2021). Thus, evaluating BFR only by metabolic stress is incomplete. For practical programming, the key question is whether the acute metabolic and perceptual responses are tolerable and whether they affect subsequent movement performance. Meta-analytic evidence indicates that, in fixed-repetition protocols, low-load BFR resistance exercise produces higher RPE and discomfort than load-matched non-BFR exercise (de Queiros et al., 2023) ,while comparisons of clinical and practical BFR show that cuff application method can further affect perceptual responses (Miller et al., 2020).

This concern is especially relevant for track and field athletes, whose training often requires rapid transitions between strength work, jumping, landing, sprinting, and technical tasks. Drop-jump (DJ) testing provides a useful way to examine short-term explosive performance and landing behavior after an exercise bout. DJ height reflects rebound performance and stretch-shortening cycle function, whereas loading rate reflects how rapidly vertical ground reaction force rises during landing and may indicate changes in impact attenuation. Center of pressure (COP) sample entropy adds a nonlinear description of the regularity of COP behavior during landing, although entropy outcomes require careful interpretation because they depend on signal length, parameter selection, and the analyzed time window (Richman and Moorman, 2000; Nigg and Wakeling, 2001; Ramdani et al., 2009; Markwick et al., 2015). Fatigue-related changes in drop-jump or single-leg landing kinetics provide a useful reference point for interpreting immediate post-exercise landing outcomes (Watanabe et al., 2016; Lisee et al., 2020; Ramirez-Campillo et al., 2023).

Despite extensive BFR research, several gaps remain. First, many studies focus on training adaptations, vascular responses, or perceptual outcomes in isolation, rather than integrating metabolic, perceptual, and post-exercise movement outcomes in the same experimental design. Second, cuff-width studies often emphasize AOP or hemodynamics, but fewer studies examine whether cuff width also changes subjective load and subsequent landing-related outcomes. Third, for trained athletes, the immediate practical issue is whether different cuff widths alter acute metabolic and perceptual responses together with temporary post-exercise performance changes. Addressing these gaps requires a within-subject design that reduces between-participant variability and directly compares cuff-width conditions under standardized exercise procedures.

Therefore, the present study used a randomized crossover design to compare No-BFR, 6cm-BFR, and 12cm-BFR conditions during low-load Smith-machine half-squat exercise in trained male long-jump athletes. Blood lactate was used to characterize metabolic stress; RPE and NPRS were used to characterize perceptual and pain responses; and DJ height, loading rate, and COP sample entropy were used to examine immediate post-exercise landing outcomes. We hypothesized that BFR would increase metabolic and perceptual responses compared with No-BFR, that the 12cm-BFR condition would produce greater acute responses than the 6cm-BFR condition, and that BFR would be associated with short-term changes in DJ-derived landing outcomes. The broader aim was to clarify how cuff width may be used more intelligently in BFR prescription for athletic populations.

## 2 Materials and methods

### 2.1 Participants

Twenty-two trained male long jump athletes were recruited from Capital University of Physical Education and Sports. Although the authors were affiliated with the School of Education, Beijing Sport University, participant recruitment and all experimental procedures were conducted at Capital University of Physical Education and Sports, which served as the host institution for data collection. Participants were classified as trained athletes according to the international participant-classification framework (McKay et al., 2022). Inclusion criteria were at least 5 years of systematic training experience, a minimum training frequency of 4 sessions per week, and no lower-limb musculoskeletal injury within the previous 6 months or known cardiovascular disease.

Prior to participation, all individuals provided written informed consent. Because participant recruitment and all experimental procedures were conducted at Capital University of Physical Education and Sports, the study was reviewed and approved by the Ethics Committee of Capital University of Physical Education and Sports (Approval No. 2025A424). All procedures were conducted in accordance with the Declaration of Helsinki.

Participant characteristics (Mean ± SD) were as follows: age 21.3 ± 1.4 years, height 178.6 ± 5.7 cm, body mass 71.8 ± 6.3 kg, Smith-machine half-squat 1RM 134.5 ± 18.7 kg, and relative strength 1.87 ± 0.21 × body mass.

Sample size estimation was performed *a priori* using G*Power 3.1. Based on a medium effect size (f = 0.25), α = 0.05, power (1 − β) = 0.80, a repeated-measures correlation of 0.5, three conditions, and 2–4 repeated measurements depending on the outcome, the required minimum sample size was estimated to be 18 participants.

All 22 participants (100%) completed all three crossover conditions and every prescribed repetition in the 30-15-15–15 sequence without external assistance or failure to maintain the prescribed cadence; all participants were included in the final analysis.

### 2.2 Experimental design

**Figure 1:**
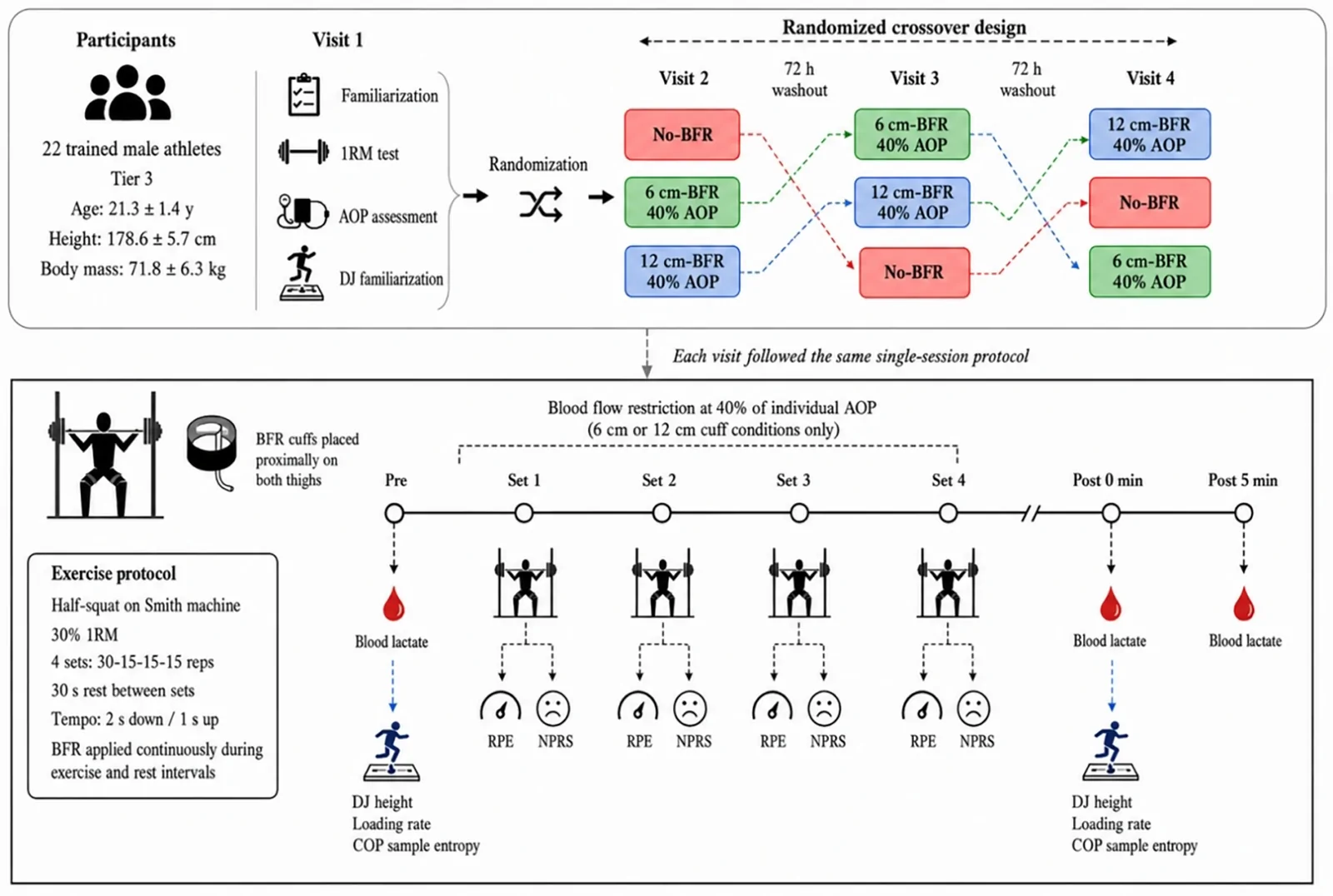
Experimental design and exercise protocol timeline. AOP, arterial occlusion pressure; BFR, blood flow restriction; DJ, drop jump; COP, center of pressure; RPE, rating of perceived exertion; NPRS, numerical pain rating scale.

A randomized crossover design was employed. Each participant completed three experimental conditions in counterbalanced order: No-BFR, 6cm-BFR, and 12cm-BFR. Sessions were separated by 72 h to reduce potential carryover effects. Participants attended a familiarization session before formal testing to standardize the half-squat protocol, DJ task, and subjective rating procedures (**Figure 1**).

#### 2.2.1 Blood flow restriction device, cuff characteristics, and arterial occlusion pressure assessment

BFR pressure was delivered using a KAATSU Master pneumatic pressure-control unit (KAATSU, Japan) connected to two third-party reusable pneumatic thigh cuffs (BOLRZO, Shenzhen, China). The cuffs had identical external lengths of 85 cm but differed in nominal external width: 6 cm for the narrow cuff and 12 cm for the medium-width cuff. The corresponding single-chamber inflatable bladders measured 60 × 5 cm and 60 × 11 cm, respectively. Both cuffs consisted of a non-elastic woven nylon outer sleeve, an integrated thermoplastic polyurethane bladder, a hook-and-loop fastening mechanism, and identical pneumatic tubing and connectors. The cuffs were straight, non-contoured, and non-segmented; thus, cuff width represented the principal constructional difference between the two conditions. The KAATSU Master was used as the inflation and pressure-maintenance source, whereas actual cuff pressure was independently verified in mmHg using a calibrated NIBP-1010 non-invasive blood pressure simulator connected to the pneumatic circuit through a compatible T-shaped pneumatic connector and tubing assembly. Proprietary Standard KAATSU Units (SKU) were not treated as mmHg. The same connection configuration was used for both cuff widths.

AOP was determined independently for the left and right lower limbs and separately for the 6-cm and 12-cm cuffs. The order of limb and cuff-width assessment was randomized. Before assessment, participants rested quietly in the supine position for 10 min with both knees extended and the lower-limb musculature relaxed. AOP was assessed during Visit 1, before the three randomized crossover exercise sessions, and was not reassessed immediately before each individual exercise session.

For each assessment, the cuff was positioned at the most proximal portion of the thigh. The upper edge of the cuff was placed approximately 2 cm distal to the inguinal crease anteriorly and aligned with the inferior gluteal fold posteriorly. The cuff was oriented perpendicular to the longitudinal axis of the thigh, and the pneumatic tube was positioned laterally.

Distal arterial pulse was detected over the posterior tibial artery, immediately posterior to the medial malleolus, using a Dopplex D900 handheld vascular Doppler equipped with a VP8XS 8-MHz vascular probe (Huntleigh Healthcare Ltd., Cardiff, United Kingdom). Actual cuff pressure was independently monitored in mmHg using an NIBP-1010 non-invasive blood pressure simulator connected to the pneumatic circuit through a compatible T-shaped pneumatic connector and tubing assembly between the KAATSU Master pressure-control unit and the pneumatic cuff. Cuff pressure was progressively increased from 0 mmHg at approximately 10 mmHg·s^-1^ while the arterial signal was continuously monitored. AOP was defined as the lowest cuff pressure at which the distal Doppler signal was completely absent for at least 5 s.

Each limb- and cuff-specific AOP assessment was performed twice. Between repeated measurements using the same cuff width on the same limb, the cuff was not removed or repositioned; it remained in place and was completely deflated for a 2-min recovery interval. When the cuff width was changed, the first cuff was removed and the alternative cuff was positioned using the same anatomical landmarks and orientation described above. When the first two measurements differed by more than 10 mmHg, a third assessment was performed. The mean of the two closest measurements was used as the final AOP. Exercise pressure was set at 40% of the corresponding limb- and cuff-specific AOP. The same physical cuff used to determine AOP was used during the corresponding exercise condition. Detailed cuff specifications are provided in Attachment 1, **Supplementary Table 1**; cuff-specific AOP values and standardized BFR reporting information are provided in **Supplementary Tables 2**, **S3**; photographs of both cuff widths are presented in **Supplementary Figure 1**; and the pneumatic connection configuration is illustrated in **Supplementary Figure 2**.

During each experimental session, the cuffs were positioned bilaterally using the same anatomical landmarks and orientation employed during AOP assessment. Placement and pressure setting were performed by the same investigator across all crossover sessions. The prescribed limb-specific pressure was continuously monitored and maintained throughout all four exercise sets and the three 30-s inter-set rest periods. Pressure was released immediately after completion of the final set. During exercise and inter-set rest, pressure was verified using the external pressure manometer.

#### 2.2.2 Experimental control

All experiments were conducted in a controlled laboratory environment (temperature: 22–24 °C; relative humidity: 40–60%). To minimize circadian variation, all testing sessions were performed at the same time of day (09:00–11:00).

Three trained investigators conducted all sessions. W.C. was responsible for overall protocol supervision and coordination; Y.H. was responsible for cuff placement, AOP assessment, pressure application, and pressure monitoring; and Y.C. was responsible for physiological measurements and data recording, including blood lactate sampling and the administration of RPE and NPRS ratings. The same investigators retained their assigned roles throughout all sessions to ensure consistency across testing conditions.

#### 2.2.3 Testing procedure

Each session followed the same standardized sequence. Participants completed a 5–10 min warm-up consisting of light aerobic activity and dynamic stretching, followed by baseline DJ testing for DJ height, loading rate, and COP sample entropy. Participants then performed the assigned low-load half-squat exercise protocol. RPE and NPRS were recorded immediately after each set, and blood lactate was measured at Pre, Post 0 min, and Post 5 min. Post-exercise DJ testing was performed 3 min after cuff release using the same procedures as baseline.

### 2.3 Blood flow restriction exercise protocol

Participants performed low-load Smith-machine half squats in each experimental condition. Feet were positioned approximately shoulder width apart, and the barbell was placed across the upper back. Participants were instructed to maintain the same foot position, depth, and trunk posture across conditions.

The exercise intensity was set at 30% of individual 1RM. The exercise consisted of four sets with a repetition sequence of 30, 15, 15, and 15 repetitions, a commonly used BFR resistance-exercise structure (Patterson et al., 2019). A fixed 30 s rest interval was allowed between sets. Movement tempo was standardized at approximately 2 s for the descending phase and 1 s for the ascending phase. The 1RM was determined once during familiarization, and the same absolute load corresponding to 30% of baseline 1RM was used across all three crossover conditions.

In the 6cm-BFR and 12cm-BFR conditions, cuff pressure followed the individualized procedure described above. The No-BFR condition was performed without cuff application. The same standardized technical instructions were provided in all conditions, and participants were encouraged to complete all prescribed repetitions while maintaining proper technique.

### 2.4 Measurements

#### 2.4.1 One-repetition maximum test

The one-repetition maximum (1RM) for the Smith machine squat was determined following standardized strength assessment guidelines (Kraemer and Ratamess, 2004). Participants completed a 5–10 min warm-up consisting of light aerobic activity and dynamic stretching. This was followed by 5–10 repetitions at 40–60% of the estimated 1RM and 3–5 repetitions at 60–80%.

The initial load for formal testing was set at approximately 90% of the estimated 1RM and progressively increased until failure. A rest interval of 3–5 min was provided between attempts to minimize fatigue. The highest load successfully lifted with correct technique was recorded as the 1RM. The test was terminated if the participant failed to complete the lift or if movement technique was compromised. All assessments were supervised by trained investigators, with standardized verbal encouragement provided throughout.

#### 2.4.2 Drop jump test

Lower-limb explosive performance was assessed using a DJ test from a fixed 30 cm platform. This height is commonly used in reactive-strength and stretch-shortening-cycle assessment (Markwick et al., 2015; Healy et al., 2018).

All trials were conducted on a three-dimensional force platform at a sampling frequency of 1000 Hz. Participants stepped forward from the 30 cm platform, landed with both feet simultaneously, and immediately performed a maximal vertical jump while minimizing ground contact time. Hands were placed on the hips throughout the trial to remove arm-swing effects. Contact time was defined from initial ground contact to take-off during the rebound phase, and flight time was defined from take-off to the subsequent landing.

DJ height was calculated from flight time derived from the vertical ground reaction force signal. Loading rate was calculated as the rate of increase in vertical ground reaction force during the initial impact phase, from initial contact to the first impact peak. COP sample entropy was calculated to quantify the regularity and complexity of COP behavior during landing. Additional reliability information for COP sample entropy and cuff-specific AOP determination is provided in Attachment 1, **Supplementary Table 7**.

Each participant completed three valid trials, and the mean value was used for analysis. Trials were repeated if improper technique or loss of balance occurred. The post-exercise DJ assessment was performed 3 min after completion of the exercise protocol. This standardized interval was predefined to represent an early acute post-exercise assessment window, allowing sufficient time for cuff release and test preparation while remaining close to the exercise bout to capture transient alterations in explosive performance and landing biomechanics.

#### 2.4.3 Blood lactate, RPE, and NPRS

Capillary blood lactate concentration was measured to evaluate exercise-induced metabolic stress. Blood samples (~20 μL) were collected from the fingertip using sterile disposable lancets and analyzed immediately using a portable lactate analyzer (Lactate Scout+, EKF Diagnostics, Germany), which has demonstrated acceptable validity for field-based lactate assessment (Tanner et al., 2010).

Measurements were obtained at three time points: pre-exercise (Pre), immediately post-exercise (Post 0 min), and 5 min post-exercise (Post 5 min). Before sampling, the fingertip was cleaned with alcohol and dried. The first drop of blood was discarded, and the second drop was used for analysis.

RPE was assessed immediately after each set using the Borg 6–20 scale, where 6 indicates no exertion and 20 indicates maximal exertion. Pain was assessed immediately after each set using an 11-point NPRS, where 0 indicates no pain and 10 indicates the worst imaginable pain (Hawker et al., 2011).

### 2.5 Data processing

Ground reaction force (GRF) data were collected at 1000 Hz and processed using a fourth-order zero-lag Butterworth low-pass filter with a cutoff frequency of 50 Hz. Initial contact was defined as the point at which vertical GRF exceeded 10 N.

DJ height was calculated using the flight-time method. Loading rate was determined from the vertical GRF signal during the initial impact phase, from initial contact to the first impact peak. Values were normalized to body weight and reported as BW/s.

The COP trajectory was derived from force-platform data. COP sample entropy was calculated during the DJ landing phase using the resultant COP displacement. The analysis window was defined from initial contact to take-off. COP data were processed using the same filtering procedure as the GRF data. SampEn was computed with embedding dimension m = 2 and tolerance r = 0.2 × SD of each individual time series. The same processing procedure was applied to all participants, conditions, and time points.

Each participant performed three valid DJ trials per condition and time point, and the mean value was used for subsequent analysis. Trials with technical errors or unstable landing were excluded and repeated.

### 2.6 Statistical analysis

Statistical analyses were performed using SPSS version 26.0 (IBM Corp., Armonk, NY, USA). Normality was assessed using the Shapiro-Wilk test. Sphericity was assessed using Mauchly’s test, and the Greenhouse-Geisser correction was applied when sphericity was violated. Descriptive data are presented as Mean ± SD.

Two-way repeated-measures ANOVA was conducted to examine within-subject effects of condition and time or set. Blood lactate was analyzed using a 3 condition (No-BFR, 6cm-BFR, 12cm-BFR) × 3 time (Pre, Post 0 min, Post 5 min) repeated-measures ANOVA. RPE and NPRS were analyzed using a 3 condition × 4 set (Set 1, Set 2, Set 3, Set 4) repeated-measures ANOVA. DJ height, loading rate, and COP sample entropy were analyzed using a 3 condition × 2 time (Pre, Post) repeated-measures ANOVA.

Bonferroni-adjusted *post hoc* comparisons were used for pairwise analyses. Partial eta squared (ηp²) was reported as the effect-size estimate, with thresholds of 0.01, 0.06, and 0.14 interpreted as small, medium, and large effects. Statistical significance was set at α = 0.05. Supplementary analyses of cuff-specific AOP differences and measurement repeatability, together with within-session DJ reliability and force-platform signal-quality assessments, are provided in Attachment 1 (**Supplementary Tables 2**, **4**, **5**).

To examine whether greater metabolic and perceptual stress was associated with a greater post-exercise landing-performance cost, exploratory repeated-measures correlations were conducted across the three experimental conditions. The immediate blood-lactate response was calculated as the change from Pre to Post 0 min. Because RPE and NPRS were recorded after each exercise set, the Set 4 values were selected as the perceptual indices most temporally proximal to cuff release and the post-exercise DJ assessment. DJ-height decrement was calculated as Pre minus Post, such that a larger positive value represented a greater reduction in jump performance. Changes in loading rate and COP sample entropy were calculated as Post minus Pre, with larger positive values representing greater post-exercise increases. Participant identity was treated as the repeated factor. Repeated-measures correlation coefficients (r_rm) and 95% confidence intervals were reported. Because nine associations were examined, p values were adjusted using the Holm sequential Bonferroni procedure. Statistical significance was determined using the adjusted p value, with α = 0.05.

## 3 Results

### 3.1 Blood lactate

**Figure 2:**
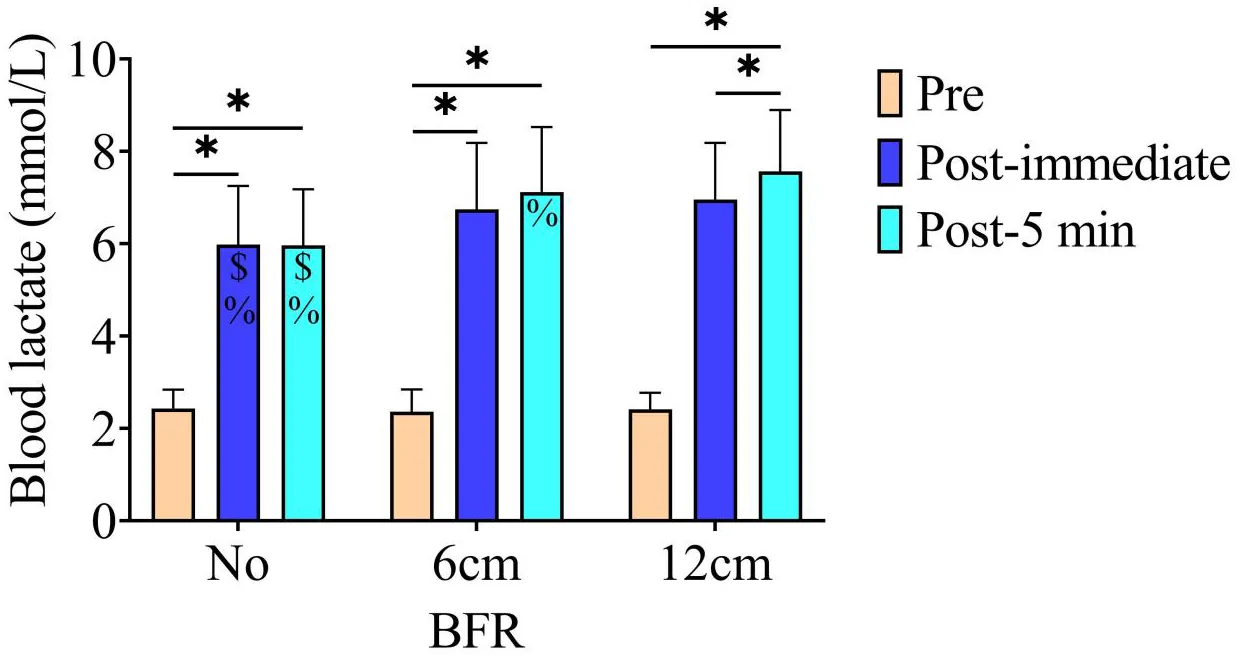
Blood lactate before exercise, immediately after exercise, and 5 min after exercise under No-BFR, 6-cm BFR, and 12-cm BFR. *Significant within-condition bracketed comparison; $significantly different from 6-cm BFR at the same time point; %significantly different from 12-cm BFR at the same time point. When $ and % appear together above the same bar, the marked value differs significantly from both BFR conditions at that time point. All pairwise comparisons were Bonferroni-adjusted (adjusted p < 0.05).

Blood lactate results are presented in **Figure 2**. Baseline values were comparable across conditions (No-BFR: 2.43 ± 0.40 mmol/L; 6-cm BFR: 2.36 ± 0.48 mmol/L; 12-cm BFR: 2.41 ± 0.36 mmol/L). Following the intervention, blood lactate increased markedly in all conditions, with higher values observed in the BFR conditions.

Two-way repeated-measures ANOVA revealed significant main effects of time, F(1.31, 27.47) = 449.832, p < 0.001, ηp² = 0.955, and condition, F(1.29, 27.10) = 31.967, p < 0.001, ηp² = 0.604, as well as a significant condition × time interaction, F(2.24, 47.12) = 14.101, p < 0.001, ηp² = 0.402.

Bonferroni-adjusted *post hoc* analyses indicated that immediately post-exercise, blood lactate was significantly higher in the 6-cm BFR (adjusted p = 0.008) and 12-cm BFR (adjusted p < 0.001) conditions than in the No-BFR condition. At 5 min post-exercise, blood lactate remained significantly higher in both the 6-cm BFR and 12-cm BFR conditions than in the No-BFR condition (both adjusted p < 0.001), and was significantly higher in the 12-cm BFR condition than in the 6-cm BFR condition (adjusted p = 0.047).

Within-condition comparisons showed that blood lactate significantly increased from baseline to both immediately post-exercise and 5 min post-exercise in all three conditions (all adjusted p < 0.001). In the 12-cm BFR condition, blood lactate at 5 min post-exercise was also significantly higher than immediately post-exercise (adjusted p = 0.012).

### 3.2 RPE

**Figure 3:**
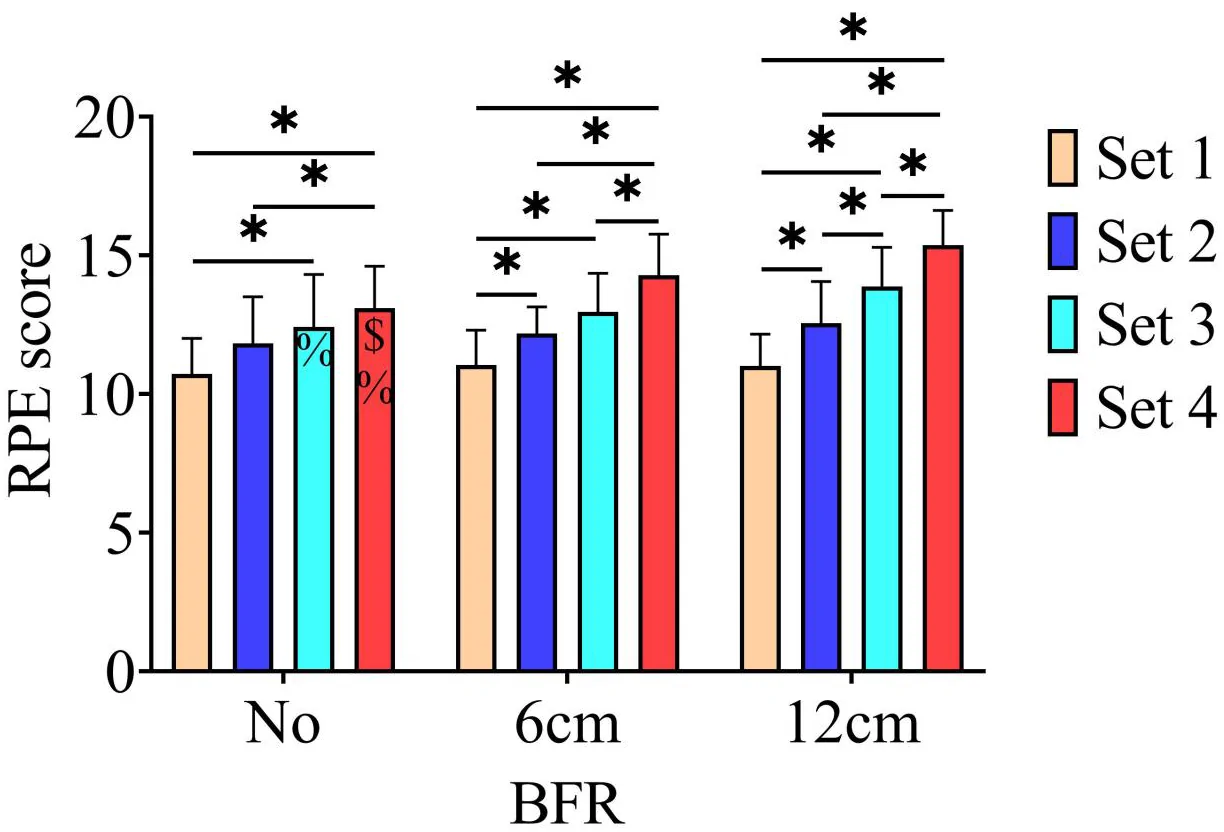
RPE across four sets under No-BFR, 6cm-BFR, and 12cm-BFR. *significant bracketed comparison within condition (p<0.05); $different from 6cm-BFR at the same set (p<0.05); %different from 12cm-BFR at the same set (p<0.05). All pairwise comparisons were Bonferroni-adjusted.

RPE results are presented in **Figure 3**. At Set 1, RPE values were comparable across conditions, with values of 10.73 ± 1.28 for No-BFR, 11.05 ± 1.25 for 6-cm BFR, and 11.00 ± 1.15 for 12-cm BFR. RPE progressively increased across sets in all three conditions.

Two-way repeated-measures ANOVA revealed significant main effects of set, F(2.44, 51.25) = 69.783, p < 0.001, ηp² = 0.769, and condition, F(1.51, 31.70) = 16.262, p < 0.001, ηp² = 0.436. The condition × set interaction was not significant, F(3.83, 80.47) = 2.077, p = 0.094, ηp² = 0.090.

Bonferroni-adjusted comparisons between conditions showed that, at Set 3, RPE was significantly higher in the 12-cm BFR condition than in the No-BFR condition (adjusted p = 0.025). At Set 4, RPE was significantly higher in both the 6-cm BFR condition (adjusted p = 0.018) and the 12-cm BFR condition (adjusted p < 0.001) than in the No-BFR condition. No significant differences among conditions were observed at Sets 1 or 2, and all remaining between-condition comparisons were not significant (adjusted p > 0.05).

Within the No-BFR condition, RPE at Set 3 was significantly higher than at Set 1 (adjusted p = 0.012). RPE at Set 4 was also significantly higher than at Set 1 (adjusted p = 0.002) and Set 2 (adjusted p = 0.016). No significant differences were observed between Sets 1 and 2, Sets 2 and 3, or Sets 3 and 4 (adjusted p > 0.05).

Within the 6-cm BFR condition, RPE was significantly higher at Set 2 (adjusted p = 0.007), Set 3 (adjusted p = 0.005), and Set 4 (adjusted p < 0.001) than at Set 1. RPE at Set 4 was also significantly higher than at Set 2 (adjusted p < 0.001) and Set 3 (adjusted p = 0.011). The difference between Sets 2 and 3 was not significant (adjusted p = 0.188).

Within the 12-cm BFR condition, RPE at Set 2 was significantly higher than at Set 1 (adjusted p = 0.002). RPE at Set 3 was significantly higher than at Set 1 (adjusted p < 0.001) and Set 2 (adjusted p = 0.017). RPE at Set 4 was significantly higher than at Set 1 (adjusted p < 0.001), Set 2 (adjusted p < 0.001), and Set 3 (adjusted p = 0.002).

### 3.3 NPRS

**Figure 4:**
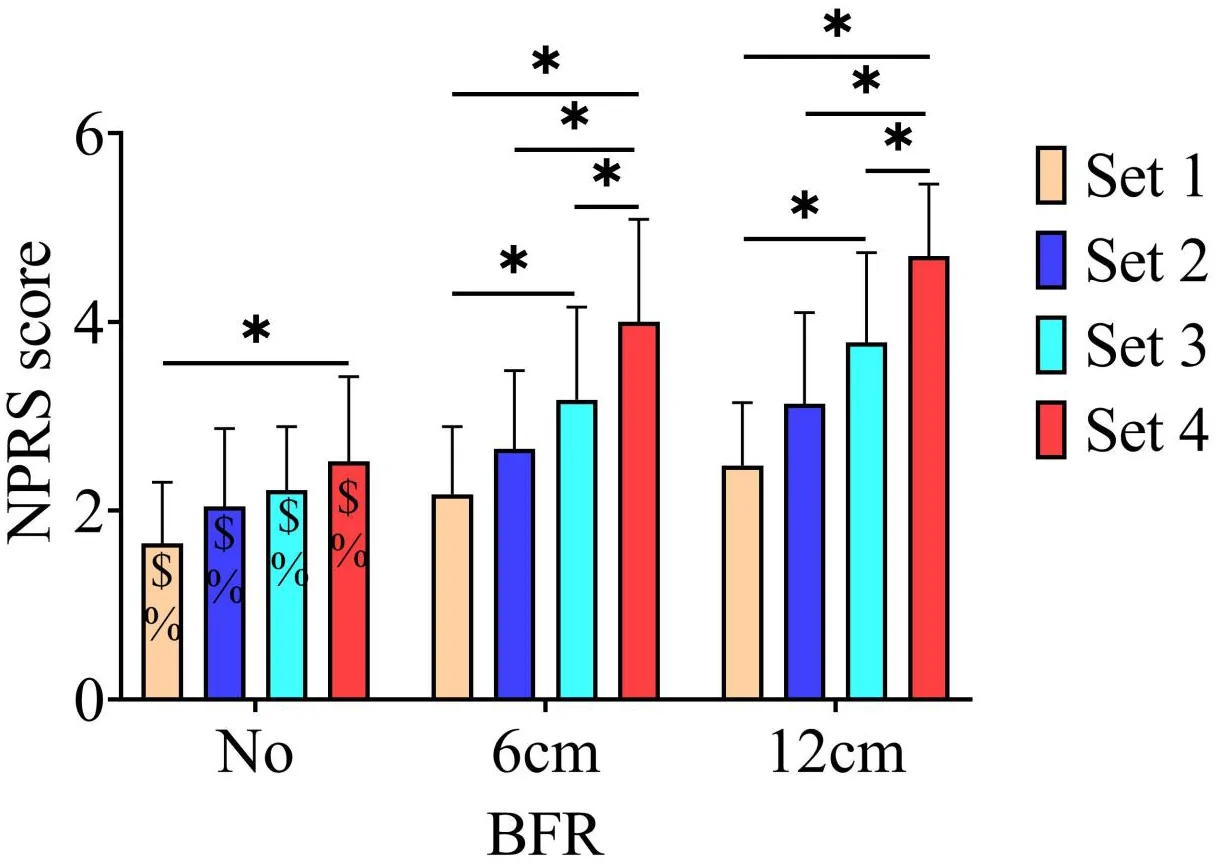
NPRS across four sets under No-BFR, 6-cm BFR, and 12-cm BFR. *Significant within-condition bracketed comparison; $significantly different from 6-cm BFR at the same set; %significantly different from 12-cm BFR at the same set. When $ and % appear together above the same bar, the marked value differs significantly from both BFR conditions at that set. All pairwise comparisons were Bonferroni-adjusted (adjusted p < 0.05).

NPRS results are presented in **Figure 4**. Following Set 1, values were 1.65 ± 0.65, 2.17 ± 0.72, and 2.48 ± 0.67 for the No-BFR, 6-cm BFR, and 12-cm BFR conditions, respectively. After Set 2, the corresponding values were 2.04 ± 0.82, 2.65 ± 0.83, and 3.13 ± 0.97. Following Set 3, NPRS increased to 2.22 ± 0.67, 3.17 ± 0.98, and 3.78 ± 0.95, and after Set 4, values reached 2.52 ± 0.90, 4.00 ± 1.09, and 4.70 ± 0.76 for the No-BFR, 6-cm BFR, and 12-cm BFR conditions, respectively.

Repeated-measures ANOVA revealed significant main effects of set, F(2.47, 51.80) = 36.378, p < 0.001, ηp² = 0.623, and condition, F(1.75, 36.79) = 39.574, p < 0.001, ηp² = 0.643, as well as a significant condition × set interaction, F(4.34, 91.04) = 4.839, p = 0.001, ηp² = 0.180.

Bonferroni-adjusted *post hoc* analyses indicated that NPRS values were significantly higher in both BFR conditions than in No-BFR at Set 1 (6-cm BFR: adjusted p = 0.003; 12-cm BFR: adjusted p = 0.002), Set 2 (adjusted p = 0.038 and adjusted p < 0.001, respectively), Set 3 (adjusted p = 0.014 and adjusted p < 0.001, respectively), and Set 4 (both adjusted p < 0.001). Within No-BFR, NPRS at Set 4 was significantly higher than at Set 1 (adjusted p = 0.037). In the 6-cm BFR condition, NPRS values at Sets 3 and 4 were significantly higher than at Set 1 (both adjusted p < 0.001), and Set 4 was higher than Sets 2 and 3 (adjusted p < 0.001 and adjusted p = 0.023, respectively). In the 12-cm BFR condition, NPRS values at Sets 3 and 4 were significantly higher than at Set 1, and Set 4 was significantly higher than Sets 2 and 3 (all adjusted p < 0.001).

### 3.4 DJ height

**Figure 5:**
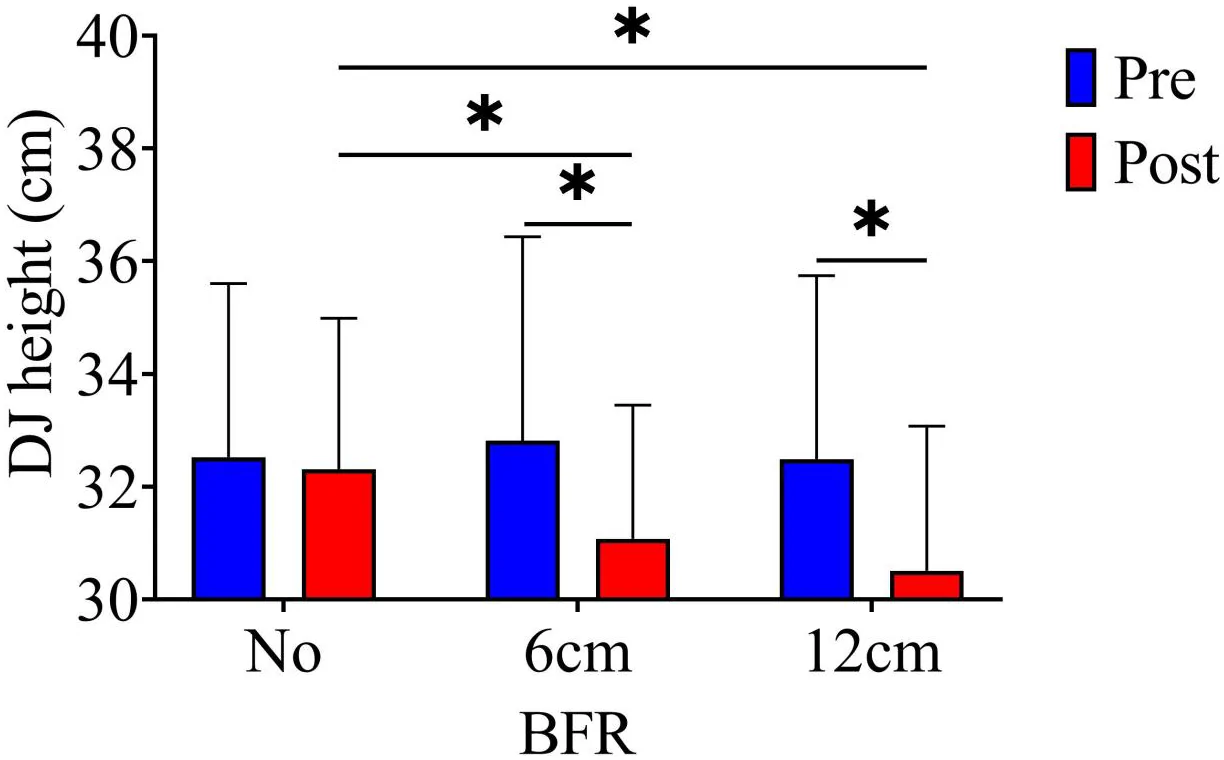
DJ height before and after exercise under No-BFR, 6cm-BFR, and 12cm-BFR. *significant bracketed comparison (p<0.05). All pairwise comparisons were Bonferroni-adjusted.

DJ height results are presented in **Figure 5**. At Pre, values were 32.52 ± 3.08 cm, 32.82 ± 3.62 cm, and 32.49 ± 3.26 cm for the No-BFR, 6-cm BFR, and 12-cm BFR conditions, respectively. At Post, values were 32.31 ± 2.68 cm, 31.07 ± 2.37 cm, and 30.51 ± 2.57 cm for the No-BFR, 6-cm BFR, and 12-cm BFR conditions, respectively.

Repeated-measures ANOVA revealed significant main effects of time, F(1, 21) = 35.485, p < 0.001, ηp² = 0.628, and condition, F(1.49, 31.36) = 20.182, p < 0.001, ηp² = 0.490, as well as a significant condition × time interaction, F(1.76, 37.04) = 20.163, p < 0.001, ηp² = 0.490.

Bonferroni-adjusted *post hoc* analyses showed that at Post, DJ height was significantly lower in both the 6-cm BFR and 12-cm BFR conditions than in the No-BFR condition (both adjusted p < 0.001). Within-condition comparisons showed that DJ height was significantly lower at Post than at Pre in both BFR conditions (both adjusted p < 0.001).

Expressed as within-participant percentage change, DJ height changed by −0.5 ± 2.4% in No-BFR, −4.9 ± 4.4% in 6-cm BFR, and −5.9 ± 4.1% in 12-cm BFR. Thus, the two BFR conditions were associated with an average post-exercise DJ-height reduction of approximately 5–6%, whereas the mean change under No-BFR was less than 1%.

No serious or unexpected adverse events occurred during the experimental sessions, and no participant required medical evaluation or treatment.

### 3.5 Loading rate

**Figure 6:**
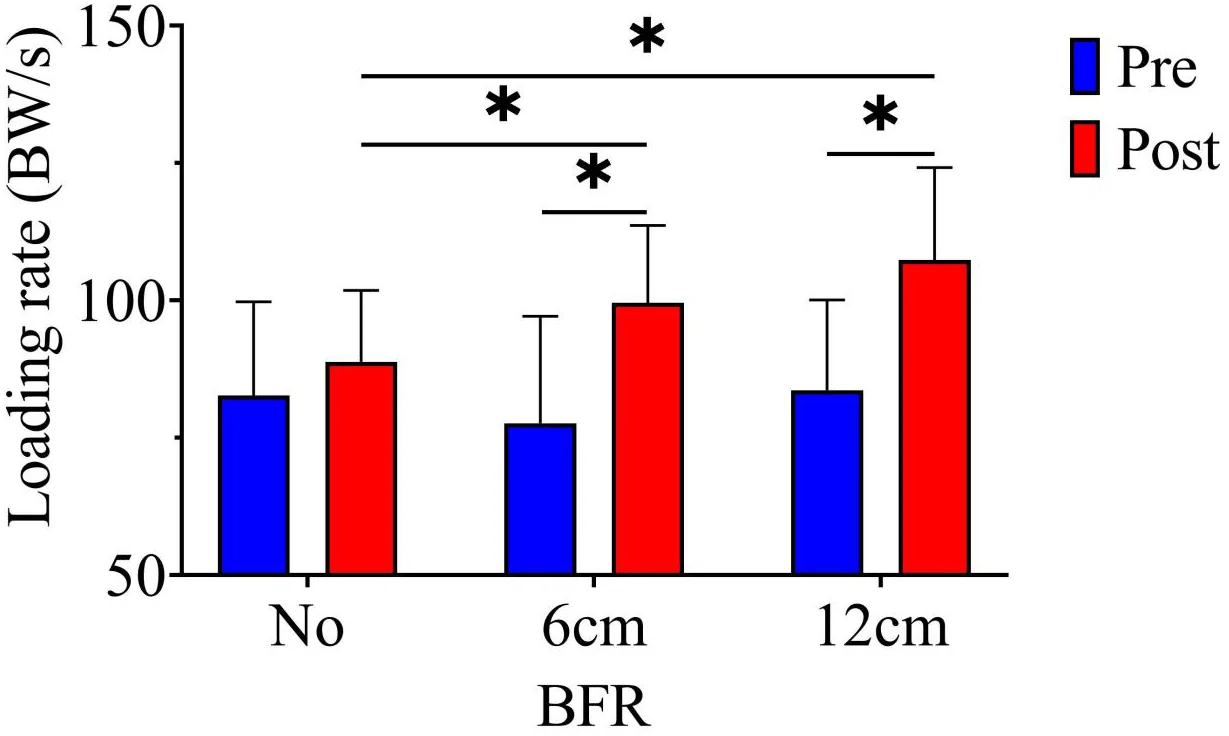
Loading rate before and after exercise under No-BFR, 6cm-BFR, and 12cm-BFR. *significant bracketed comparison (p<0.05). All pairwise comparisons were Bonferroni-adjusted.

Loading rate results are presented in **Figure 6**. At Pre, values were 82.67 ± 17.04 BW/s, 77.64 ± 19.46 BW/s, and 83.65 ± 16.41 BW/s for the No-BFR, 6-cm BFR, and 12-cm BFR conditions, respectively. At Post, values were 88.82 ± 12.99 BW/s, 99.53 ± 14.10 BW/s, and 107.35 ± 16.81 BW/s for the No-BFR, 6-cm BFR, and 12-cm BFR conditions, respectively.

Repeated-measures ANOVA revealed a significant main effect of time, F(1, 21) = 72.248, p < 0.001, ηp² = 0.775, a non-significant main effect of condition, F(1.83, 38.47) = 2.973, p = 0.067, ηp² = 0.124, and a significant condition × time interaction, F(1.87, 39.31) = 3.850, p = 0.032, ηp² = 0.155.

Bonferroni-adjusted *post hoc* analyses showed that at Post, loading rate was significantly higher in the 6-cm BFR (adjusted p = 0.034) and 12-cm BFR (adjusted p = 0.007) conditions than in the No-BFR condition. Within-condition comparisons showed that loading rate was significantly higher at Post than at Pre in both BFR conditions (both adjusted p < 0.001).

### 3.6 COP sample entropy

**Figure 7:**
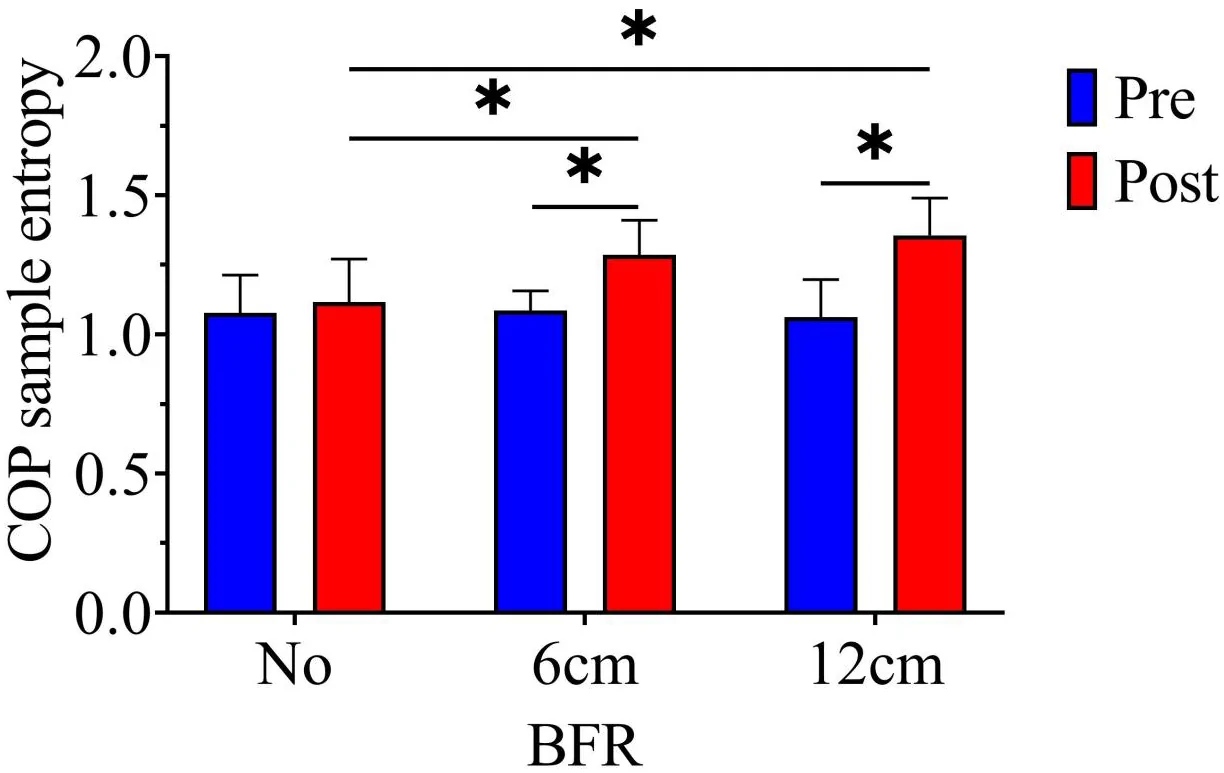
COP sample entropy before and after exercise under No-BFR, 6cm-BFR, and 12cm-BFR. *significant bracketed comparison (p<0.05). All pairwise comparisons were Bonferroni-adjusted.

COP sample entropy results are presented in **Figure 7**. At Pre, values were 1.08 ± 0.13, 1.09 ± 0.07, and 1.06 ± 0.14 for the No-BFR, 6-cm BFR, and 12-cm BFR conditions, respectively. At Post, values were 1.12 ± 0.16, 1.29 ± 0.13, and 1.36 ± 0.13 for the No-BFR, 6-cm BFR, and 12-cm BFR conditions, respectively.

Repeated-measures ANOVA revealed significant main effects of time, F(1, 21) = 73.763, p < 0.001, ηp² = 0.778, and condition, F(1.77, 37.13) = 16.543, p < 0.001, ηp² = 0.441, as well as a significant condition × time interaction, F(1.51, 31.65) = 7.521, p = 0.004, ηp² = 0.264.

Bonferroni-adjusted *post hoc* analyses showed that at Post, COP sample entropy was significantly higher in both the 6-cm BFR and 12-cm BFR conditions than in the No-BFR condition (both adjusted p < 0.001). Within-condition comparisons showed that COP sample entropy was significantly higher at Post than at Pre in both BFR conditions (both adjusted p < 0.001).

### 3.7 Associations between metabolic, perceptual, and DJ-derived responses

Exploratory repeated-measures correlations were conducted to determine whether greater metabolic and perceptual stress across the three experimental conditions was associated with a greater post-exercise landing-performance cost.

A greater immediate increase in blood lactate was associated with a larger DJ-height decrement (r_rm = 0.392, 95% CI [0.112, 0.615], Holm-adjusted p = 0.038) and a greater increase in loading rate (r_rm = 0.505, 95% CI [0.248, 0.695], adjusted p = 0.003). The association between the blood-lactate response and the increase in COP sample entropy did not remain statistically significant after correction for multiple comparisons (r_rm = 0.364, 95% CI [0.079, 0.594], adjusted p = 0.056).

Higher Set 4 RPE was associated with a larger DJ-height decrement (r_rm = 0.413, 95% CI [0.136, 0.630], adjusted p = 0.034). Its associations with increases in loading rate (r_rm = 0.341, 95% CI [0.053, 0.577], adjusted p = 0.065) and COP sample entropy (r_rm = 0.308, 95% CI [0.016, 0.551], adjusted p = 0.079) did not remain significant following Holm correction.

Higher Set 4 NPRS was strongly associated with a larger DJ-height decrement (r_rm = 0.637, 95% CI [0.422, 0.784], adjusted p < 0.001) and was also associated with a greater increase in COP sample entropy (r_rm = 0.413, 95% CI [0.136, 0.630], adjusted p = 0.034). The association between Set 4 NPRS and loading-rate change was not significant (r_rm = 0.260, 95% CI [−0.036, 0.515], adjusted p = 0.084). Attachment 1 (**Supplementary Table 6**). A structured summary of the principal outcomes and findings is provided in Attachment 1, **Supplementary Table 8**.

## 4 Discussion

This study examined whether cuff width modifies acute responses to low-load BFR exercise in trained male long-jump athletes. The main finding was that both BFR conditions increased blood lactate, RPE, and NPRS compared with No-BFR, with the 12cm-BFR condition generally producing the largest acute metabolic and perceptual responses. Post-exercise DJ height decreased, whereas loading rate and COP sample entropy increased after BFR. These results suggest that cuff width can influence acute metabolic and perceptual responses together with short-term post-exercise movement changes. The study therefore contributes to BFR prescription by showing that cuff width is not merely an equipment characteristic, but a factor that may shape acute metabolic, perceptual, and immediate post-exercise landing responses.

### 4.1 Metabolic stress: why cuff width may matter

The blood lactate response supports the interpretation that BFR increased acute metabolic stress during low-load resistance exercise. This interpretation is consistent with the broader BFR literature, which suggests that restricted venous return and partial limitation of arterial inflow can create an intramuscular environment characterized by reduced oxygen availability and greater metabolite accumulation (Suga et al., 2009; Patterson et al., 2019). The practical importance of this response is that greater acute metabolic responses were observed while the external resistance remained at 30% 1RM, which may be valuable when heavy loading is undesirable or when training volume must be accumulated without excessive joint stress.

The interpretation of the present results should account for the specific cuff configuration used in this study. The KAATSU Master served as the pneumatic pressure-control unit, whereas the tested 6-cm and 12-cm cuffs were third-party BOLRZO cuffs. The cuffs had the same external length, non-elastic woven-nylon sleeve, single thermoplastic-polyurethane bladder, straight non-contoured construction, and pneumatic connections; their principal difference was the external and inflatable-bladder width (60 × 5 cm versus 60 × 11 cm). Wider cuffs generally require lower absolute pressures to achieve arterial occlusion because pressure is distributed over a larger contact area (Loenneke et al., 2012). Moreover, cuffs that differ in width and construction should not be assumed to be mechanically or hemodynamically interchangeable, because cuff configuration can alter the pressure transmitted to the limb and the resulting blood-flow response (Mouser et al., 2017). Accordingly, the greater lactate response, the generally higher RPE and NPRS, and the larger post-exercise landing changes observed with the 12 cm cuff are best interpreted as a cuff-configuration effect under cuff-specific 40% AOP prescription, rather than as evidence that cuff width alone universally produces a stronger physiological or performance response.

At the same time, the metabolic finding must be interpreted within the limits of the measured variables. Blood lactate is a useful marker of systemic and local metabolic stress, but it does not directly measure muscle oxygenation, venous pooling, arterial inflow, or intramuscular pressure. Therefore, the present data support the conclusion that the 12cm-BFR condition increased metabolic response more than the 6cm-BFR condition, but they do not prove the precise vascular mechanism.

### 4.2 Perceptual responses, pain, and athlete tolerance

The RPE and NPRS results indicate that BFR was accompanied by higher perceived exertion and pain than the No-BFR condition. This response is expected because BFR exercise can increase sensations of pressure, burning, fatigue, and discomfort, particularly as metabolites accumulate and cuff compression is maintained across repeated sets (Spitz et al., 2020; Spitz et al., 2021). In the present protocol, pressure remained applied during both exercise and the 30-s inter-set intervals, which may have contributed to the progressive increases in RPE and NPRS across the four sets. Existing evidence indicates that BFR-related perceptual responses depend on exercise design, pressure, and cuff configuration. A meta-analysis of 30 studies found that, in fixed-repetition protocols, BFR resistance exercise produced higher RPE (SMD = 1.04, p < 0.01) and discomfort (SMD = 1.10, p < 0.01) than load-matched low-load resistance exercise without BFR (de Queiros et al., 2023). During elbow-flexion training at 80% of cuff-specific AOP, Laurentino et al. (2016) reported no significant differences in RPE or perceived pain between 5-cm and 10-cm cuffs. Similarly, Spitz et al. (2021) found no exercise-related discomfort difference between 5-cm and 12-cm cuffs when width-appropriate relative pressures were used. In contrast, Soligon et al. (2018) found that low-pressure BFR at 40–50% AOP produced lower overall RPE than BFR at 70–80% AOP and lower pain than BFR at 60–80% AOP. Miller et al. (2020) also reported greater RPE and discomfort with clinical pneumatic BFR than with practical BFR. Collectively, these findings indicate that RPE, pain, and discomfort are influenced by relative pressure, cuff configuration, application method, and exercise protocol rather than cuff width alone.

For athletes, these outcomes are practically relevant because RPE and NPRS directly quantify perceived exertion and pain experienced during the exercise bout. A protocol associated with greater discomfort may be more or less tolerable depending on the athlete and the intended session context. The 12-cm BFR condition generally showed the highest mean RPE and NPRS scores, but direct differences between the two cuff widths were not uniform across comparisons. Therefore, the present results do not indicate that cuff width alone determines perceptual responses.

Both BFR conditions elicited higher blood lactate, RPE, and NPRS responses than No-BFR while the external resistance remained at 30% 1RM. These acute responses do not by themselves demonstrate a superior training stimulus or predict greater long-term adaptation. The use of BFR when heavy loading is constrained still requires individualized screening and supervision (Hughes et al., 2017; Minniti et al., 2020). Nevertheless, the absence of a sham-cuff condition warrants caution when interpreting RPE and NPRS. Because participants could readily distinguish the No-BFR, 6-cm BFR, and 12-cm BFR conditions, blinding to cuff application and width was not feasible. Expectancy, previous experience, and heightened attention to cuff-related sensations may therefore have partly amplified the observed perceptual differences. This limitation does not invalidate the responses, but it prevents their full magnitude from being attributed exclusively to cuff width and the associated physiological and mechanical stimulus. No serious or unexpected adverse events occurred in this trained young male sample, but this short-term tolerability observation should not be generalized beyond the present population.

### 4.3 Drop-jump height and loading rate: immediate movement cost

The decrease in DJ height after BFR suggests a short-term reduction in rebound performance. This outcome is consistent with the idea that the exercise bout produced acute fatigue or discomfort that carried into the post-exercise jump task. However, the mechanism cannot be assigned to a single source. Reduced DJ height may reflect local muscular fatigue, discomfort-related inhibition, altered effort, reduced stretch-shortening cycle efficiency, or a protective adjustment in landing and take-off strategy. Because EMG and muscle oxygenation were not collected, this finding represents an acute change in post-exercise performance and does not provide direct evidence of altered neuromuscular activation.

The increase in loading rate is also practically important. Loading rate reflects how quickly force rises during landing, and higher values may indicate reduced time or capacity for impact attenuation. In athletic settings, this matters because landing mechanics are relevant to repeated jumping, sprinting, cutting, and technical drills. The fact that loading rate increased more clearly under BFR suggests that the acute cost of the method may extend beyond subjective discomfort. If a BFR bout is followed immediately by explosive or landing-intensive tasks, coaches may need to consider whether the athlete’s landing behavior has been temporarily altered.

This does not mean that BFR is unsafe or unsuitable for athletes. Rather, it suggests that timing matters. If BFR is used as a metabolic-strength accessory after high-quality explosive work, the post-exercise decrement in DJ performance may be acceptable or even irrelevant. If BFR is used before jumping, landing, sprinting, or technical sessions, the temporary changes in DJ height and loading rate may be undesirable. This is where the present study has practical value: it helps position BFR not only as a strengthening tool, but as a programming variable that must be placed carefully within the session.

### 4.4 COP sample entropy and the boundary of interpretation

COP sample entropy increased after BFR, particularly in the wider-cuff condition. SampEn is commonly used to describe the regularity or complexity of physiological time series, and higher values generally indicate a less regular signal pattern. However, entropy should not be simplified into a direct label of better or worse control. In postural and movement research, entropy values are sensitive to data length, sampling properties, filtering, selected time window, embedding dimension, tolerance, and task demands (Richman and Moorman, 2000; Ramdani et al., 2009; Lubetzky et al., 2018). For this reason, the entropy result should be interpreted as a complexity-sensitive descriptor rather than a direct performance score, consistent with methodological work on center-of-pressure entropy and postural-system adaptability (Manor et al., 2010; Gow et al., 2015).

Accordingly, BFR was associated with a change in the regularity of COP behavior during landing. This change may reflect altered sensory feedback, fatigue, discomfort, or movement strategy, but the present design cannot separate these possibilities. Together with the DJ-height and loading-rate findings, the COP SampEn result indicates that the post-exercise landing response changed in both conventional performance measures and the temporal regularity of COP behavior.

### 4.5 Why the findings matter for BFR prescription

The central practical message is that cuff width and session timing should be considered together when prescribing BFR for athletes. Both BFR conditions elicited greater acute metabolic and perceptual responses at 30% 1RM, while DJ height decreased by approximately 5-6% and loading rate increased after the BFR bouts. These findings indicate that greater acute metabolic and perceptual responses may be accompanied by a short-term cost to explosive and landing performance. Therefore, when immediate jumping, sprinting, or technical quality must be preserved, BFR may be better positioned after those high-quality tasks rather than before them.

The lower external resistance used during BFR remains practically attractive. During rehabilitation or return-to-training, it may provide a low-load lower-limb exercise option when heavy loading is temporarily limited. During periods of high training density, it may allow coaches to add local muscular stress without imposing the same external mechanical burden as another heavy resistance session. In trained athletes, BFR can also be used as an accessory method after the primary technical or explosive component of a session. Athlete-focused reviews support its broader use as an adjunct to conventional training, provided that pressure, exercise selection, and progression are individualized (Wortman et al., 2020; Yang et al., 2024).

This application is particularly relevant to track and field athletes, whose programs already include substantial mechanical stress from sprinting, jumping, landing, and resistance training. A low-load method that increases muscular demand while reducing barbell load may be useful when additional lower-limb work is desired but another high-load exposure is not. In this context, the value of BFR extends beyond general strength or hypertrophy: it permits low-load exercise to be performed with greater acute metabolic and perceptual responses than those observed without BFR, thereby providing another option for managing session content within a training week.

The choice between the 6-cm and 12-cm cuffs should remain goal dependent. A wider cuff may be considered when a stronger acute metabolic and perceptual stimulus is acceptable and no immediate explosive task follows. A narrower cuff may be preferable when comfort, tolerance, or subsequent movement quality is prioritized. Because direct between-cuff differences were not consistent for every outcome, these recommendations should not be interpreted as evidence that one cuff width is universally superior. Instead, cuff width should be treated as one component of an individualized BFR prescription that also includes relative pressure, exercise load, placement within the session, and the athlete’s current recovery status.

### 4.6 Relationship to existing literature

Previous acute cuff-width studies provide a useful, although incomplete, comparator for the present findings. Mouser et al. demonstrated that cuff width can modify lower-limb blood-flow responses, reinforcing that cuffs of different widths should not be assumed to create identical vascular conditions simply because pressure is expressed relative to an occlusion value (Mouser et al., 2018). This is consistent with the present finding that the 6-cm and 12-cm conditions produced different acute metabolic profiles despite both being prescribed at 40% of cuff-specific AOP.

The current findings also extend the available acute cuff-width literature. The cited studies primarily examined vascular or perceptual responses and did not assess immediate post-exercise drop-jump height, impact loading, or the temporal structure of center-of-pressure behavior. Accordingly, the approximately 5–6% reduction in DJ height under BFR, together with the increases in loading rate and COP sample entropy, adds a performance- and movement-related dimension to previous cuff-width research.

### 4.7 Limitations and future directions

Several limitations should be acknowledged when interpreting the present findings. The sample consisted exclusively of 22 trained young male track and field athletes, which may limit generalizability to female participants, untrained individuals, older adults, clinical populations, or athletes from other sporting disciplines. Only a single relative pressure was applied; therefore, the observed responses may differ under lower or higher percentages of arterial occlusion pressure, and the interaction between cuff width and applied pressure should not be assumed to be linear. The present study focused solely on acute responses and cannot determine whether the observed condition-dependent differences would translate into superior or clinically meaningful long-term adaptations. Although sessions were counterbalanced and separated by 72 h, formal order or carryover effects were not reported. The absence of a sham-cuff condition and the practical difficulty of blinding participants to cuff application may have influenced subjective perceptual outcomes, particularly RPE and NPRS. Moreover, because electromyography, muscle oxygenation, ultrasound-based blood-flow assessment, and direct intramuscular pressure measurements were not included, mechanistic interpretations related to neural drive, muscle activation, vascular restriction, or local tissue compression should be made cautiously. AOP was assessed in the supine position during Visit 1 rather than in the standing exercise position immediately before each experimental session. Because AOP is position dependent (de Queiros et al., 2024), the prescribed pressures may not have represented exactly 40% of the exercise-position AOP, and possible day-to-day variation was not captured. Future studies should determine AOP in the exercise-relevant position immediately before each experimental session.

Future studies should compare multiple relative pressures across cuff widths, include both male and female athletes, and examine whether acute metabolic and perceptual responses predict chronic adaptation. Combining force-platform outcomes with EMG, near-infrared spectroscopy, ultrasound blood-flow assessment, and reactive strength index measures would help determine whether post-exercise DJ changes are driven primarily by metabolic fatigue, pain, altered muscle activation, or movement strategy. Such work would strengthen the mechanistic basis for selecting cuff width in sport-specific BFR programming.

## 5 Conclusion

In conclusion, BFR increased acute metabolic and perceptual responses during low-load half-squat exercise and was accompanied by short-term changes in DJ-derived landing outcomes. The 12-cm BFR condition generally produced larger mean responses than the 6-cm BFR condition, although direct between-cuff differences were not consistent across all outcomes. These findings indicate that cuff width should be considered when interpreting acute metabolic, perceptual, and post-exercise movement responses under the tested low-load BFR protocol. The reductions in DJ height and the increases in loading rate and COP sample entropy represent immediate post-exercise changes and should not be interpreted as beneficial training effects. Because EMG, muscle oxygenation, blood-flow imaging, and intramuscular pressure were not measured, the precise mechanisms underlying these acute responses cannot be determined from the present data. These acute metabolic, perceptual, and performance responses do not demonstrate that either cuff condition provides a superior training stimulus and cannot be used to predict long-term adaptation.

## Data Availability

The raw data supporting the conclusions of this article will be made available by the authors, without undue reservation.

## References

[B15] Aniceto R. R. da Silva L. L. (2022). Practical blood flow restriction training: New methodological directions for practice and research. Sports Med. - Open 8 (1), 87. doi: 10.1186/s40798-022-00475-2 35763185 PMC9240154

[B16] Clarkson M. J. May A. K. Warmington S. A. (2020). Is there rationale for the cuff pressures prescribed for blood flow restriction exercise? A systematic review. Scandinavian J. Med. Sci. Sports 30 (8), 1318–1336. doi: 10.1111/sms.13676 32279391

[B24] de Queiros V. S. Rolnick N. Dos Santos Í.K. de França I. M. Lima R. J. Vieira J. G. . (2023). Acute effect of resistance training with blood flow restriction on perceptual responses: A systematic review and meta-analysis. Sports Health 15 (5), 673–688. doi: 10.1177/19417381221131533 36415041 PMC10467469

[B17] de Queiros V. S. Rolnick N. Kamiş O. Formiga M. F. Rocha R. F. C. Alves J. C. M. . (2024). Body position and cuff size influence lower limb arterial occlusion pressure and its predictors: Implications for standardizing the pressure applied in training with blood flow restriction. Front. Physiol. 15, 1446963. doi: 10.3389/fphys.2024.1446963 39189031 PMC11345145

[B41] Gow B. Peng C.-K. Wayne P. Ahn A. (2015). Multiscale entropy analysis of center-of-pressure dynamics in human postural control: Methodological considerations. Entropy 17, 7926–7947. doi: 10.3390/e1712784930654563

[B37] Hawker G. A. Mian S. Kendzerska T. French M. (2011). Measures of adult pain: Visual analog scale for pain (VAS pain), numeric rating scale for pain (NRS pain), McGill pain questionnaire (MPQ), short-form McGill pain questionnaire (SF-MPQ), chronic pain grade scale (CPGS), short form-36 bodily pain scale (SF-36 BPS), and measure of intermittent and constant osteoarthritis pain (ICOAP). Arthritis Care Res. 63 (Suppl. 11), S240–S252. doi: 10.1002/acr.20543 22588748

[B35] Healy R. Kenny I. C. Harrison A. J. (2018). Reactive strength index: A poor indicator of reactive strength? Int. J. Sports Physiol. Perform. 13, 802–809. doi: 10.1123/ijspp.2017-0511 29182434

[B11] Hill E. C. Rivera P. M. Proppe C. E. Gonzalez Rojas D. H. Wizenberg A. M. Keller J. L. (2022). Greater neuromuscular fatigue following low-load blood flow restriction than non-blood flow restriction resistance exercise among recreationally active men. J. Neurophysiol. 128 (1), 73–85. doi: 10.1152/jn.00028.2022 35704398 PMC9273273

[B38] Hughes L. Paton B. Rosenblatt B. Gissane C. Patterson S. D. (2017). Blood flow restriction training in clinical musculoskeletal rehabilitation: A systematic review and meta-analysis. Br. J. Sports Med. 51 (13), 1003–1011. doi: 10.1136/bjsports-2016-097071 28259850

[B14] Jessee M. B. Buckner S. L. Dankel S. J. Counts B. R. Abe T. Loenneke J. P. (2016). The influence of cuff width, sex, and race on arterial occlusion: Implications for blood flow restriction research. Sports Med. (Auckland N.Z.) 46 (6), 913–921. doi: 10.1007/s40279-016-0473-5 26820301

[B34] Kraemer W. J. Ratamess N. A. (2004). Fundamentals of resistance training: Progression and exercise prescription. Med. Sci. Sports Exercise 36, 674–688. doi: 10.1249/01.mss.0000121945.36635.61 15064596

[B20] Laurentino G. C. Loenneke J. P. Teixeira E. L. Nakajima E. Iared W. Tricoli V. (2016). The effect of cuff width on muscle adaptations after blood flow restriction training. Med. Sci. Sports Exercise 48 (5), 920–925. doi: 10.1249/MSS.0000000000000833 26656773

[B5] Li R. Chee C. S. Kamalden T. F. Ramli A. S. Yang K. (2024). Effects of blood flow restriction training on sports performance in athletes: A systematic review with meta-analysis. J. Sports Med. Phys. Fitness 64 (1), 55–65. doi: 10.23736/S0022-4707.23.15220-0 37902798

[B31] Lisee C. Birchmeier T. Yan A. Geers B. O'Hagan K. Davis C. . (2020). The relationship between vertical ground reaction force, loading rate, and sound characteristics during a single-leg landing. J. Sport Rehabil. 29 (5), 541–546. doi: 10.1123/jsr.2018-0260 31034335

[B3] Lixandrão M. E. Ugrinowitsch C. Berton R. Vechin F. C. Conceição M. S. Damas F. . (2018). Magnitude of muscle strength and mass adaptations between high-load resistance training versus low-load resistance training associated with blood-flow restriction: A systematic review and meta-analysis. Sports Med. (Auckland N.Z.) 48 (2), 361–378. doi: 10.1007/s40279-017-0795-y 29043659

[B13] Loenneke J. P. Fahs C. A. Rossow L. M. Sherk V. D. Thiebaud R. S. Abe T. . (2012). Effects of cuff width on arterial occlusion: Implications for blood flow restricted exercise. Eur. J. Appl. Physiol. 112 (8), 2903–2912. doi: 10.1007/s00421-011-2266-8 22143843 PMC4133131

[B40] Lubetzky A. V. Harel D. Lubetzky E. (2018). On the effects of signal processing on sample entropy for postural control. PloS One 13 (3), e0193460. doi: 10.1371/journal.pone.0193460 29494625 PMC5832259

[B42] Manor B. Costa M. D. Hu K. Newton E. Starobinets O. Kang H. G. . (2010). Physiological complexity and system adaptability: Evidence from postural control dynamics of older adults. J. Appl. Physiol. (Bethesda Md. 1985) 109 (6), 1786–1791. doi: 10.1152/japplphysiol.00390.2010 20947715 PMC3006415

[B26] Markwick W. J. Bird S. P. Tufano J. J. Seitz L. B. Haff G. G. (2015). The intraday reliability of the reactive strength index calculated from a drop jump in professional men's basketball. Int. J. Sports Physiol. Perform. 10 (4), 482–488. doi: 10.1123/ijspp.2014-0265 25394213

[B33] McKay A. K. A. Stellingwerff T. Smith E. S. Martin D. T. Mujika I. Goosey-Tolfrey V. L. . (2022). Defining training and performance caliber: A participant classification framework. Int. J. Sports Physiol. Perform. 17 (2), 317–331. doi: 10.1123/ijspp.2021-0451 34965513

[B25] Miller R. M. Galletti B. A. R. Koziol K. J. Freitas E. D. S. Heishman A. D. Black C. D. . (2020). Perceptual responses: Clinical versus practical blood flow restriction resistance exercise. Physiol. Behav. 227, 113137. doi: 10.1016/j.physbeh.2020.113137 32798570

[B39] Minniti M. C. Statkevich A. P. Kelly R. L. Rigsby V. P. Exline M. M. Rhon D. I. . (2020). The safety of blood flow restriction training as a therapeutic intervention for patients with musculoskeletal disorders: A systematic review. Am. J. Sports Med. 48 (7), 1773–1785. doi: 10.1177/0363546519882652 31710505

[B18] Mouser J. G. Dankel S. J. Jessee M. B. Mattocks K. T. Buckner S. L. Counts B. R. . (2017). A tale of three cuffs: The hemodynamics of blood flow restriction. Eur. J. Appl. Physiol. 117 (7), 1493–1499. doi: 10.1007/s00421-017-3644-7 28501908

[B19] Mouser J. G. Dankel S. J. Mattocks K. T. Jessee M. B. Buckner S. L. Abe T. . (2018). Blood flow restriction and cuff width: Effect on blood flow in the legs. Clin. Physiol. Funct. Imaging 38 (6), 944–948. doi: 10.1111/cpf.12504 29356291

[B8] Nalbandian M. Takeda M. (2016). Lactate as a signaling molecule that regulates exercise-induced adaptations. Biology 5 (4), 38. doi: 10.3390/biology5040038 27740597 PMC5192418

[B27] Nigg B. M. Wakeling J. M. (2001). Impact forces and muscle tuning: A new paradigm. Exercise Sport Sci. Rev. 29 (1), 37–41. doi: 10.1097/00003677-200101000-00008 11210446

[B2] Ohta H. Kurosawa H. Ikeda H. Iwase Y. Satou N. Nakamura S. (2003). Low-load resistance muscular training with moderate restriction of blood flow after anterior cruciate ligament reconstruction. Acta Orthopaedica Scandinavica 74 (1), 62–68. doi: 10.1080/00016470310013680 12635796

[B12] Patterson S. D. Hughes L. Warmington S. Burr J. Scott B. R. Owens J. . (2019). Blood flow restriction exercise: Considerations of methodology, application, and safety. Front. Physiol. 10. doi: 10.3389/fphys.2019.00533 31156448 PMC6530612

[B7] Pearson S. J. Hussain S. R. (2015). A review on the mechanisms of blood-flow restriction resistance training-induced muscle hypertrophy. Sports Med. (Auckland N.Z.) 45 (2), 187–200. doi: 10.1007/s40279-014-0264-9 25249278

[B9] Pope Z. K. Willardson J. M. Schoenfeld B. J. (2013). Exercise and blood flow restriction. J. Strength Conditioning Res. 27 (10), 2914–2926. doi: 10.1519/JSC.0b013e3182874721 23364292

[B29] Ramdani S. Seigle B. Lagarde J. Bouchara F. Bernard P. L. (2009). On the use of sample entropy to analyze human postural sway data. Med. Eng. Phys. 31, 1023–1031. doi: 10.1016/j.medengphy.2009.06.004 19608447

[B32] Ramirez-Campillo R. Thapa R. K. Afonso J. Perez-Castilla A. Bishop C. Byrne P. J. . (2023). Effects of plyometric jump training on the reactive strength index in healthy individuals across the lifespan: A systematic review with meta-analysis. Sports Med. (Auckland N.Z.) 53 (5), 1029–1053. doi: 10.1007/s40279-023-01825-0 36906633 PMC10115703

[B28] Richman J. S. Moorman J. R. (2000). Physiological time-series analysis using approximate entropy and sample entropy. Am. J. Physiol. Heart Circ. Physiol. 278, H2039–H2049. doi: 10.1152/ajpheart.2000.278.6.h2039 10843903

[B23] Soligon S. D. Lixandrão M. E. Biazon T. Angleri V. Roschel H. Libardi C. A. (2018). Lower occlusion pressure during resistance exercise with blood-flow restriction promotes lower pain and perception of exercise compared to higher occlusion pressure when the total training volume is equalized. Physiol. Int. 105 (3), 276–284. doi: 10.1556/2060.105.2018.3.18 30269562

[B22] Spitz R. W. Chatakondi R. N. Bell Z. W. Wong V. Viana R. B. Dankel S. J. . (2021). Blood flow restriction exercise: Effects of sex, cuff width, and cuff pressure on perceived lower body discomfort. Perceptual Motor Skills 128 (1), 353–374. doi: 10.1177/0031512520948295 32777996

[B21] Spitz R. W. Wong V. Bell Z. W. Viana R. B. Chatakondi R. N. Abe T. . (2020). Blood flow restricted exercise and discomfort: A review. J. Strength Conditioning Res. 36, 871–879. doi: 10.1519/jsc.0000000000003525 32058360

[B6] Suga T. Okita K. Morita N. Yokota T. Hirabayashi K. Horiuchi M. . (2009). Intramuscular metabolism during low-intensity resistance exercise with blood flow restriction. J. Appl. Physiol. (Bethesda Md. 1985) 106 (4), 1119–1124. doi: 10.1152/japplphysiol.90368.2008 19213931

[B36] Tanner R. K. Fuller K. L. Ross M. L. R. (2010). Evaluation of three portable blood lactate analysers: Lactate Pro, Lactate Scout and Lactate Plus. Eur. J. Appl. Physiol. 109 (3), 551–559. doi: 10.1007/s00421-010-1379-9 20145946

[B30] Watanabe S. Aizawa J. Shimoda M. Enomoto M. Nakamura T. Okawa A. . (2016). Effect of short-term fatigue, induced by high-intensity exercise, on the profile of the ground reaction force during single-leg anterior drop-jumps. J. Phys. Ther. Sci. 28 (12), 3371–3375. doi: 10.1589/jpts.28.3371 28174454 PMC5276763

[B10] Wernbom M. Aagaard P. (2020). Muscle fibre activation and fatigue with low-load blood flow restricted resistance exercise-an integrative physiology review. Acta Physiologica (Oxford England) 228 (1), e13302. doi: 10.1111/apha.13302 31108025

[B1] Wortman R. J. Brown S. M. Savage-Elliott I. Finley Z. J. Mulcahey M. K. (2020). Blood flow restriction training for athletes: A systematic review. Am. J. Sports Med. 49, 1938–1944. doi: 10.1177/0363546520964454 33196300

[B4] Wortman R. J. Mulcahey M. Elliot I. Finley Z. Brown S. (2021). Blood flow restriction therapy for athletes: A systematic review. Arthroscopy 37(1 Suppl.), e21. doi: 10.1016/j.arthro.2020.12.044 33196300

[B43] Yang K. Chee C. S. Abdul Kahar J. Tengku Kamalden T. F. Li R. Qian S. (2024). Effects of blood flow restriction training on physical fitness among athletes: A systematic review and meta-analysis. Sci. Rep. 14 (1), 16615. doi: 10.1038/s41598-024-67181-9 39025894 PMC11258269

