# Supplementary material for: Influence of blood flow restriction cuff width on acute metabolic, perceptual, and landing responses to low-load resistance exercise in trained long jump athletes
Source: Front Physiol. 2026 Sep 11;17:1912080. doi: 10.3389/fphys.2026.1912080 (PMC13612298; doi:10.3389/fphys.2026.1912080)
Supplement: Supplementary file 1 [file SupplementaryFile1.docx]

# Attachment1

**Table S1. Technical characteristics of the pneumatic thigh cuffs**

| **Characteristic** | **6-cm cuff** | **12-cm cuff** |
| --- | --- | --- |
| **Brand / supplier** | BOLRZO, Shenzhen, China | BOLRZO, Shenzhen, China |
| **External length** | 85 cm | 85 cm |
| **External width** | 6 cm | 12 cm |
| **Bladder length** | 60 cm | 60 cm |
| **Bladder width** | 5 cm | 11 cm |
| **Outer material** | Non-elastic woven nylon | Non-elastic woven nylon |
| **Bladder material** | TPU | TPU |
| **Bladder configuration** | Single chamber | Single chamber |
| **Profile** | Straight, non-contoured | Straight, non-contoured |
| **Segmentation** | Non-segmented | Non-segmented |
| **Fastening** | Hook-and-loop | Hook-and-loop |
| **Pneumatic connector** | Identical | Identical |

**Table S2. Cuff-specific AOP and reliability results**

| **Variable** | **6-cm cuff** | **12-cm cuff** |
| --- | --- | --- |
| **AOP, mmHg** | 244.2 ± 21.6 | 180.3 ± 16.8 |
| **Exercise pressure at 40% AOP, mmHg** | 97.7 ± 8.6 | 72.1 ± 6.7 |
| **ICC(A,1), 95% CI** | 0.936 (0.889–0.965) | 0.914 (0.852–0.951) |
| **ICC(A,2)** | 0.967 | 0.955 |
| **CV, %** | 2.6 | 2.9 |
| **SEM, mmHg** | 5.5 | 4.9 |
| **MDC95, mmHg** | 15.2 | 13.6 |
| **Third assessments, n** | 3 | 2 |

**Table S3. Blood Flow Restriction Reporting Checklist**

Purpose：This appendix summarizes the blood flow restriction apparatus, cuff construction, arterial occlusion pressure determination, pressure prescription, exercise protocol, and safety reporting used in the present study to improve methodological transparency and reproducibility.

| **Reporting item** | **Description reported in the present study** |
| --- | --- |
| **Pressure-control device** | KAATSU Master pneumatic pressure-control unit (KAATSU, Japan) |
| **Cuff manufacturer / supplier** | BOLRZO (Shenzhen, China) |
| **Cuff type** | Reusable pneumatic thigh cuffs |
| **Pneumatic connection** | The KAATSU Master pressure-control unit, the NIBP-1010 pressure-verification device, and the pneumatic cuff were connected through a compatible T-shaped pneumatic connector and tubing assembly. |
| **Connection consistency** | The same connection configuration was used for the 6-cm and 12-cm cuff conditions. |
| **Pressure reference** | External mmHg readings obtained from the NIBP-1010; proprietary SKU values were not interpreted as mmHg. |
| **Apparatus figure** | The apparatus configuration and pneumatic connection are illustrated in Supplementary Figure S2. |
| **Number of cuff widths** | Two (6 cm and 12 cm) |
| **External cuff length** | 85 cm for both cuffs |
| **External cuff width** | 6 cm and 12 cm |
| **Inflatable bladder length** | 60 cm for both cuffs |
| **Inflatable bladder width** | 5 cm for the 6-cm cuff and 11 cm for the 12-cm cuff |
| **Outer sleeve material** | Non-elastic woven nylon |
| **Inflatable bladder material** | Thermoplastic polyurethane (TPU) |
| **Bladder configuration** | Single-chamber pneumatic bladder |
| **Cuff profile** | Straight, non-contoured |
| **Segmentation** | Non-segmented |
| **Fastening system** | Hook-and-loop closure |
| **Pneumatic connection** | Identical pneumatic tubing and connectors for both cuffs |
| **Body region** | Bilateral proximal thighs |
| **Cuff placement** | The upper edge was positioned approximately 2 cm distal to the inguinal crease anteriorly and aligned with the inferior gluteal fold posteriorly; the cuff was perpendicular to the longitudinal axis of the thigh, with the pneumatic tube positioned laterally. |
| **Body position during AOP assessment** | Supine after 10 min of quiet rest, with both knees fully extended and the lower-limb musculature relaxed |
| **Artery monitored** | Posterior tibial artery, immediately posterior to the medial malleolus |
| **Arterial pulse detection device** | Dopplex D900 handheld vascular Doppler equipped with a VP8XS 8-MHz vascular probe (Huntleigh Healthcare Ltd., Cardiff, United Kingdom) |
| **AOP determination criterion** | The lowest cuff pressure at which the distal Doppler signal was completely absent for at least 5 s |
| **Pressure increase rate** | Approximately 10 mmHg·s⁻¹ |
| **Number of AOP measurements** | Two measurements per limb and cuff width |
| **Repeat criterion** | A third measurement was performed when the first two measurements differed by more than 10 mmHg |
| **Final AOP value** | Mean of the two closest measurements |
| **Exercise pressure prescription** | 40% of the corresponding limb- and cuff-specific AOP |
| **Mean bilateral AOP: 6-cm cuff** | 244.2 ± 21.6 mmHg |
| **Mean bilateral AOP: 12-cm cuff** | 180.3 ± 16.8 mmHg |
| **Exercise pressure: 6-cm cuff** | 97.7 ± 8.6 mmHg |
| **Exercise pressure: 12-cm cuff** | 72.1 ± 6.7 mmHg |
| **Pressure maintenance** | The prescribed limb-specific pressure was continuously monitored and maintained throughout all four exercise sets and the three 30-s inter-set rest periods |
| **Pressure release** | Immediately after completion of the fourth exercise set |
| **AOP reliability statistics** | ICC(A,1), ICC(A,2), coefficient of variation, standard error of measurement, and MDC95 are reported in Supplementary Table S2 |
| **Operator consistency** | All AOP assessments, cuff placement, and pressure setting were performed by the same trained investigator |
| **Exercise mode** | Smith-machine half squat |
| **Exercise intensity** | 30% of the baseline 1RM |
| **Sets and repetitions** | Four sets: 30, 15, 15, and 15 repetitions |
| **Inter-set recovery** | 30 s |
| **Inter-session washout** | At least 72 h |
| **Adverse events** | No serious or unexpected adverse events occurred during the experimental sessions. Transient cuff-related pressure and discomfort were captured using the NPRS and resolved after cuff deflation. |

**Abbreviations:** AOP, arterial occlusion pressure; BFR, blood flow restriction; ICC, intraclass correlation coefficient; MDC95, minimal detectable change at the 95% confidence level; NPRS, Numerical Pain Rating Scale; TPU, thermoplastic polyurethane; 1RM, one-repetition maximum.

**Reporting statement.** This appendix is provided to improve methodological transparency and reproducibility in accordance with recent recommendations emphasizing that blood flow restriction methods and apparatus should be reported in sufficient technical detail (PMID: 39919806).

**Table S4. Within-session reliability and force-signal quality at the 3-min post-exercise DJ assessment**

| **Variable** | **ICC(A,3)** | **95% CI** | **CV (%)** | **Interpretation** |
| --- | --- | --- | --- | --- |
| DJ height | 0.948 | 0.918–0.968 | 4.3 | Excellent |
| Loading rate | 0.891 | 0.832–0.930 | 8.9 | Good |
| COP sample entropy | 0.842 | 0.763–0.898 | 11.7 | Good |

**Table S5. Unloaded force-platform baseline quality-control results**

| **Metric** | **Mean ± SD** | **Maximum** | **Acceptance criterion** |
| --- | --- | --- | --- |
| Absolute baseline offset, N | 0.7 ± 1.0 | 4.2 | ≤5 N |
| Baseline SD, N | 0.8 ± 0.4 | 1.9 | ≤2 N |

**Table S6. Indicator Correlation Analysis**

| **`Predictor** | **DJ-derived change** | **r_rm** | **95% CI** | **Raw p** | **Holm-adjusted p** |
| --- | --- | --- | --- | --- | --- |
| ΔLactate (Post 0−Pre) | DJ-height decrement | 0.392 | 0.112–0.615 | 0.008 | 0.038 |
| ΔLactate (Post 0−Pre) | Loading-rate increase | 0.505 | 0.248–0.695 | <0.001 | 0.003 |
| ΔLactate (Post 0−Pre) | COP SampEn increase | 0.364 | 0.079–0.594 | 0.014 | 0.056 |
| Set 4 RPE | DJ-height decrement | 0.413 | 0.136–0.630 | 0.005 | 0.034 |
| Set 4 RPE | Loading-rate increase | 0.341 | 0.053–0.577 | 0.022 | 0.065 |
| Set 4 RPE | COP SampEn increase | 0.308 | 0.016–0.551 | 0.040 | 0.079 |
| Set 4 NPRS | DJ-height decrement | 0.637 | 0.422–0.784 | <0.001 | <0.001 |
| Set 4 NPRS | Loading-rate increase | 0.260 | −0.036–0.515 | 0.084 | 0.084 |
| Set 4 NPRS | COP SampEn increase | 0.413 | 0.136–0.630 | 0.005 | 0.034 |

**Table S7 measurement reliability**

| **Measure** | **Reliability model** | **ICC** | **95% CI** | **CV** | **SEM** | **MDC95** |
| --- | --- | --- | --- | --- | --- | --- |
| COP SampEn, three-trial mean | ICC(A,3) | 0.842 | 0.763–0.898 | 11.7% | — | — |
| AOP, 6-cm cuff | ICC(A,1) / ICC(A,2) | 0.936 / 0.967 | 0.889–0.965* | 2.6% | 5.5 mmHg | 15.2 mmHg |
| AOP, 12-cm cuff | ICC(A,1) / ICC(A,2) | 0.914 / 0.955 | 0.852–0.951* | 2.9% | 4.9 mmHg | 13.6 mmHg |

**Table S8. Summary of key acute outcomes**

| **Outcome** | **No-BFR** | **6cm-BFR** | **12cm-BFR** | **Condition × time/set** | **Key interpretation** |
| --- | --- | --- | --- | --- | --- |
| **Blood lactate, mmol/L** | Pre 2.43±0.41 Post0 5.98±1.27 Post5 5.96±1.22 | Pre 2.36±0.49 Post0 6.74±1.44 Post5 7.12±1.41 | Pre 2.41±0.36 Post0 6.96±1.22 Post5 7.56±1.33 | F(2.24,47.12)=14.101 p<0.001; ηp²=0.402 | Both BFR > No-BFR; 12cm > 6cm at Post5 (p=0.047) |
| **RPE** | Set1 10.73±1.28 Set4 13.09±1.51 | Set1 11.05±1.25 Set4 14.27±1.49 | Set1 11.00±1.15 Set4 15.36±1.26 | F(4.66,97.94)=2.271 p=0.058; ηp²=0.098 | Set and condition main effects; interaction not significant |
| **NPRS** | Set1 1.68±0.65 Set4 2.45±0.86 | Set1 2.23±0.69 Set4 4.00±1.11 | Set1 2.55±0.60 Set4 4.73±0.77 | F(4.34,91.04)=4.878 p<0.001; ηp²=0.189 | Both BFR > No-BFR at all sets; 6cm vs 12cm not significant |
| **DJ height, cm** | Pre 32.52±3.08 Post 32.31±2.68 −0.5±2.4% | Pre 32.82±3.62 Post 31.07±2.37 −4.9±4.4% | Pre 32.49±3.26 Post 30.51±2.57 −5.9±4.1% | F(1.76,37.04)=20.163 p<0.001; ηp²=0.490 | Both BFR decreased and were lower than No-BFR at Post |
| **Loading rate, BW/s** | Pre 82.67±17.04 Post 88.82±12.99 | Pre 77.64±19.46 Post 99.53±14.10 | Pre 83.65±16.41 Post 107.35±16.81 | F(1.87,39.31)=3.850 p=0.032; ηp²=0.155 | Both BFR increased and were higher than No-BFR at Post |
| **COP sample entropy** | Pre 1.08±0.14 Post 1.12±0.16 | Pre 1.09±0.07 Post 1.29±0.13 | Pre 1.06±0.14 Post 1.36±0.13 | F(1.51,31.65)=7.580 p=0.004; ηp²=0.265 | Both BFR increased and were higher than No-BFR at Post |

**Note.** Values are mean ± SD. Post0, immediately post-exercise; Post5, 5 min post-exercise; RPE, rating of perceived exertion; NPRS, Numerical Pain Rating Scale; DJ, drop jump; COP, center of pressure. Exact Bonferroni-adjusted pairwise p values are reported in the Results. Descriptive values were recalculated from the uploaded participant-level dataset (n = 22).

Supplementary Figure S1. Pneumatic thigh cuffs used in the study. (A) 6-cm cuff; (B) 12-cm cuff.

Supplementary Figure S2. Schematic representation of the pneumatic connection used in the study. (A) Overall apparatus configuration. The KAATSU Master served as the inflation and pressure-maintenance source, whereas the NIBP-1010 was connected to the cuff line through a compatible T-shaped pneumatic connector for independent pressure verification in mmHg. (B) Connector/tubing assembly showing the three branches connected to the KAATSU Master outlet tubing, the NIBP-1010 pressure-measurement port, and the pneumatic thigh cuff. (C) The 6-cm and 12-cm cuffs; the same connection configuration was used for both cuff-width conditions. The figure is a schematic reconstruction prepared during revision for methodological clarification and is not a contemporaneous photograph of an experimental session.
